# Nonlinear Hyper-Viscoelastic Constitutive Modeling and PRF Parameter Identification of Rubber Materials

**DOI:** 10.3390/polym18141687

**Published:** 2026-07-08

**Authors:** Mingkuan Wang, Jiaheng Yao, Long Zhang, Ang Gao, Enchao Zhang, Shimin Zhang, Xiaoxiao Zhu

**Affiliations:** 1 College of Mechanical and Transportation Engineering, China University of Petroleum (Beijing), Beijing 102249, China; mkwang_cup@outlook.com (M.W.); gd13453993334@163.com (J.Y.); 15232711962@163.com (L.Z.); olcdvau03@126.com (A.G.); zhangenchao195617@163.com (E.Z.); smzhang@cup.edu.cn (S.Z.); 2 College of Safety and Ocean Engineering, China University of Petroleum (Beijing), Beijing 102249, China; 3 Key Laboratory of Oil and Gas Production Safety and Emergency Technology, Ministry of Emergency Management, Beijing 102249, China

**Keywords:** rubber materials, hyper-viscoelastic constitutive model, parallel rheological framework, Prony series, stress relaxation

## Abstract

To accurately characterize the nonlinear hyper-viscoelastic mechanical behavior of rubber materials under large deformation and stress relaxation conditions, this study investigates fluororubber (FKM) and hydrogenated nitrile rubber (HNBR) with different hardness levels through uniaxial mechanical tests and stress relaxation experiments. A constitutive parameter identification method based on hyperelastic models and the parallel rheological framework (PRF) model is established. First, several representative hyperelastic models, including the Neo-Hookean, Mooney–Rivlin, Yeoh, Ogden, Arruda–Boyce, and Van der Waals models, are comparatively evaluated. The results show that the Ogden model with (N = 3) provides the highest fitting accuracy for the large-deformation responses of FKM and HNBR with different hardness levels, with coefficients of determination (*R*^2^) ranging from 0.9879 to 0.9948. Subsequently, the Prony series parameters are identified from the stress relaxation data and converted into the initial parameters of the linear PRF model. To overcome the limitations of the linear PRF model in predicting nonlinear relaxation behavior, the PRF parameters are further optimized using the Isight data matching method combined with the Hooke–Jeeves algorithm. Finite element validation demonstrates that the optimized nonlinear PRF model can accurately predict the stress relaxation behavior of both FKM and HNBR. The mean absolute percentage errors of FKM60, FKM70, and FKM80 are 2.67%, 1.57%, and 2.56%, respectively, while those of HNBR60, HNBR70, and HNBR80 are 2.16%, 2.72%, and 2.58%, respectively. These results indicate that the combination of the Ogden (N = 3) hyperelastic model and the optimized nonlinear PRF model can effectively describe the large-deformation and time-dependent viscoelastic responses of rubber materials, providing a reliable constitutive modeling basis for finite element analysis and parameter calibration of rubber sealing structures.

## 1. Introduction

The accurate determination of constitutive equations is fundamental to rubber material design, process optimization, and the structural design and performance prediction of rubber products. Rubber materials exhibit pronounced nonlinearity, viscoelasticity, and path-dependent mechanical behavior. Their stress response is not only closely related to strain level, temperature, and loading rate, but is also affected by loading history, stress relaxation, hysteretic energy dissipation, and deformation-induced effects such as the Mullins effect. Therefore, establishing a constitutive model capable of simultaneously describing large-deformation nonlinear elasticity and time-/rate-dependent viscoelasticity has long been a central issue in the mechanics of rubber materials.

Current studies on rubber constitutive modeling mainly focus on hyperelastic models, viscoelastic models, and coupled hyper-viscoelastic models. Bai et al. [1] systematically reviewed several commonly used macroscopic hyperelastic constitutive models, including the Mooney–Rivlin, Neo-Hookean, Yeoh, Ogden, Arruda–Boyce, Van der Waals, and HyperFoam models, and derived their stress–strain relationships under uniaxial tension, equi-biaxial tension, pure shear, and volumetric compression, providing a theoretical basis for parameter fitting of rubber materials under large deformation. Dal et al. [2] reviewed the applicability of isotropic hyperelastic constitutive models for rubber-like materials and pointed out that different models exhibit significant differences in predictive capability depending on deformation mode, strain range, and material type. He et al. [3] further compared 85 hyperelastic constitutive models for both unfilled rubber and highly filled rubber nanocomposites, emphasizing the critical influence of model selection on the accuracy of finite element simulations. Han et al. [4], based on Treloar’s classical experimental data, investigated complete constitutive relationships of hyperelastic materials under typical deformation modes such as uniaxial tension, equi-biaxial tension, and pure shear, providing useful guidance for constructing rubber hyperelastic models under multiple loading paths.

However, purely hyperelastic models are essentially equilibrium elastic models. Although they can describe the nonlinear elastic response of rubber materials under large deformation, they cannot capture time-dependent behaviors such as strain-rate effects, stress relaxation, creep, and hysteretic energy dissipation under dynamic loading. To address this limitation, Wei et al. [5] developed a visco-hyperelastic constitutive model based on a modified Mooney–Rivlin model and a strain-rate-dependent Maxwell model, and fitted experimental data under medium-to-high strain-rate uniaxial tension and compression. Their results showed that the proposed model could effectively describe the rate-dependent mechanical response of rubber. Wang et al. [6] conducted quasi-static and SHPB dynamic compression tests on silicone rubber, analyzed its compressive mechanical behavior over a wide strain-rate range, and established a five-parameter constitutive equation incorporating strain-rate effects, demonstrating the significant rate sensitivity of silicone rubber under dynamic compression. Zhang et al. [7] investigated SiO_2_ particle-reinforced silicone rubber composites and developed a nonlinear hyper-viscoelastic constitutive model based on the Ogden strain energy density function and a rate-dependent relaxation time function. Their model could predict dynamic stress–strain responses over a broad strain-rate range while reducing the number of parameters required in conventional Prony series models. Liu et al. [8] performed uniaxial and biaxial cyclic tensile tests and stress relaxation experiments on ethylene propylene diene monomer rubber and established a visco-hyperelastic constitutive model capable of describing stress-softening behavior, providing a method for predicting nonlinear responses of rubber materials under cyclic loading.

In addition to strain-rate effects, temperature and Mullins effects are also important factors influencing the constitutive response of rubber. Yao et al. [9] investigated the temperature dependence of the hyperelastic behavior of natural rubber and filled rubber and compared the predictive capabilities of different hyperelastic models for temperature-dependent stress–strain responses. Their results indicated that temperature variations significantly affect both hyperelastic material parameters and model accuracy. Fazekas and Goda [10] established a rubber constitutive model incorporating the Mullins effect, residual strain, and time-temperature dependence, providing a theoretical basis for describing softening, residual deformation, and hysteresis in filled rubber under cyclic loading. Li and Peng [11] proposed a thermo-hyper-viscoelastic constitutive model for high-damping rubber by considering temperature effects and a Mullins damage function. They identified the model parameters stepwise using a nonlinear least-squares method, thereby improving the prediction of mechanical responses under complex loading conditions. Zhu et al. [12] developed a visco-hyperelastic constitutive model for special damping silicone rubber by decomposing the constitutive response into a parallel-coupled system consisting of a hyperelastic model and a viscoelastic model based on a modified Zener model. Through quasi-static tensile and stress relaxation tests, they verified the applicability of the coupled visco-hyperelastic model in describing the mechanical response of damping rubber.

In recent years, nonlinear viscoelastic models based on multi-network parallel frameworks have been increasingly applied to rubber constitutive modeling. Among them, the Bergström–Boyce model and the parallel rheological framework (PRF) model introduce an equilibrium network and multiple nonequilibrium viscoelastic networks, enabling the simultaneous description of equilibrium hyperelastic response and time-/rate-dependent behavior. Kayacı and Kaya [13] conducted cyclic shear/compression and stress relaxation tests on carbon-black-filled natural rubber, using the Arruda–Boyce model to describe the equilibrium response and the Bergström–Boyce model to predict transient viscoelastic behavior. Their results indicated that this approach can describe long-term relaxation behavior without relying entirely on Prony series. Michels et al. [14] applied nonlinear viscoelastic material models in Abaqus to shrinkage and warpage analyses of blow-molded parts and demonstrated that the PRF model can improve prediction accuracy for nonlinear viscoelastic materials. However, when multiple parallel networks are introduced, the number of material parameters increases substantially, making parameter calibration and optimization more challenging.

In summary, previous studies have advanced rubber constitutive modeling from the perspectives of hyperelastic model selection, strain-rate-dependent viscoelastic modeling, temperature and Mullins effect characterization, and multi-network viscoelastic frameworks. Nevertheless, several limitations remain. First, a single hyperelastic model cannot describe the time-dependent and loading-history-dependent behaviors of rubber. Second, conventional linear viscoelastic models are generally suitable for small-deformation conditions and are insufficient for accurately characterizing nonlinear viscoelastic responses under large deformation. Third, although the PRF model has strong capability in describing nonlinear viscoelasticity, it involves multiple material parameters, and its parameter identification process is relatively complex. Therefore, how to efficiently and accurately determine PRF constitutive parameters based on experimental data remains an important issue. In this study, the nonlinear hyper-viscoelastic mechanical response of rubber materials is investigated. A constitutive modeling framework combining hyperelastic models and the parallel rheological framework (PRF) is established, with particular emphasis on identifying PRF material parameters from experimental data. Therefore, the main question addressed in this study is how to accurately characterize the nonlinear hyper-viscoelastic response of rubber materials and efficiently identify the corresponding PRF parameters based on experimental data. The proposed method aims to provide a theoretical basis for finite element analysis and structural optimization of rubber products, especially dynamically loaded components such as sealing elements, vibration isolators, damping components, and tires.

## 2. Materials and Experimental Methods

### 2.1. Uniaxial Mechanical Tests

**Figure 1:**
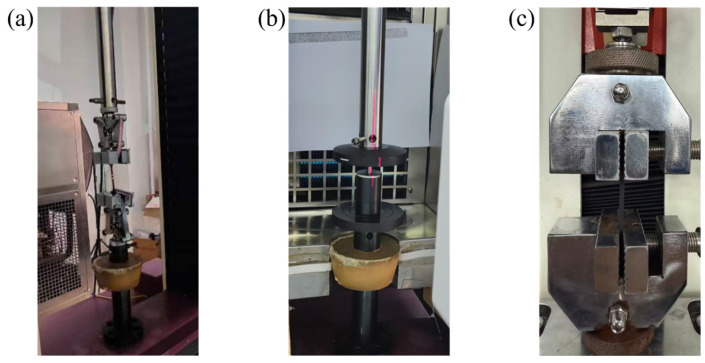
Experimental procedures: (**a**) uniaxial tensile test; (**b**) uniaxial compression test; (**c**) stress relaxation test.

To accurately characterize the large-deformation mechanical behavior of rubber materials, uniaxial tensile and uniaxial compression tests were conducted on rubber specimens using an electronic universal testing machine at China University of Petroleum (Beijing), Beijing, China. The corresponding stress–strain data were obtained to provide the experimental basis for subsequent constitutive model parameter identification. The experimental setups are shown in Figure 1a,b.

### 2.2. Stress Relaxation Test

**Figure 2:**
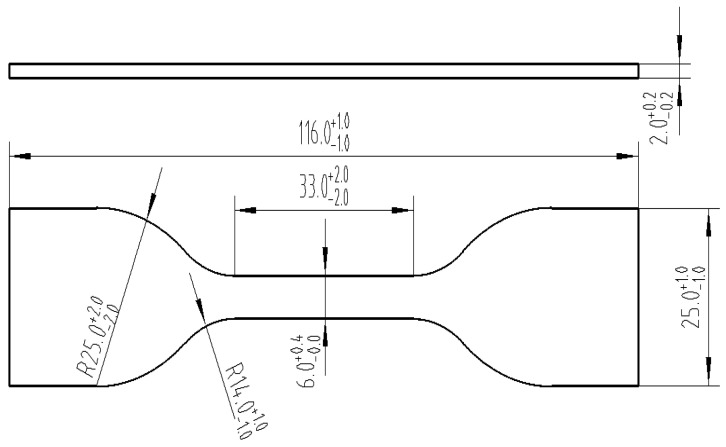
Dimensions of the stress relaxation specimen.

The tensile stress relaxation test was selected to characterize the viscoelastic behavior of the rubber specimens, as shown in Figure 1c. Dumbbell-shaped specimens were used in this study, and the specimen dimensions are shown in Figure 2.

The stress relaxation test procedure was as follows. Both ends of the dumbbell-shaped rubber specimen were firmly clamped in the grips of the universal testing machine, ensuring that the longitudinal axis of the specimen was aligned with the tensile direction and that no initial twisting or misalignment occurred. The testing machine was leveled and the grips were calibrated according to the mechanical adjustment requirements for compression stress relaxation testing. Subsequently, the specimen was freely suspended in a standard laboratory environment at (25 ± 2 °C) for 16 h to sufficiently release internal residual stresses.

During the formal test, the specimen was stretched according to Method A. First, the specimen was stretched at a rate of 50 mm/min to a gauge strain of 100%, corresponding to a gauge length extension of 25 mm. The specimen was then held at this constant strain for 5 min and subsequently unloaded to zero stress. The above “stretching–holding–unloading” procedure was repeated three times to eliminate the instantaneous elastic effect and stabilize the mechanical response of the specimen. After the preconditioning process, the specimen was again stretched at a rate of 50 mm/min to a gauge strain of 100% and then held at the constant strain for 30 min. During the holding stage, the stress decay curve with time was recorded. After completion of the holding stage, the specimen was slowly unloaded to zero stress at a rate of 1 mm/min and then allowed to stand for 30 min. Finally, the residual deformation in the gauge section was measured, the residual elongation was calculated, and the final stress relaxation curve and residual elongation data were recorded.

## 3. Constitutive Modeling of Hyper-Viscoelastic Rubber Materials

### 3.1. Elasticity Theory of Elastomers

Due to their crosslinked macromolecular network structure, elastomers can withstand large strains and exhibit pronounced nonlinear deformation behavior. From the perspective of molecular chain network theory, an elastomer is composed of randomly oriented long-chain molecules, and the chain segments between adjacent crosslinking points can be approximately regarded as freely jointed chains. When the number of chain segments is *n* and the length of each segment is *l*, the radial probability distribution of the end-to-end distance *r* can be expressed as:

(1) $$\begin{array}{c} P\left(r\right)\;=\;4\pi r^{2}{\left(\frac{3}{2\pi nl^{2}}\right)}^{\frac{3}{2}}\exp\left(-\frac{3r^{2}}{2nl^{2}}\right) \end{array}$$

where *r* is the end-to-end distance, *n* is the number of links in the molecular chain, and *l* is the length of each link.

The root-mean-square end-to-end distance *(L*_0_) can be determined from the mean-square value of the end-to-end distance, expressed as:

(2) $$L_{0}\;=\;{\left(r^{2}\right)}^{\frac{1}{2}}\;=\;{\left(nl^{2}\right)}^{\frac{1}{2}}\;=\;\sqrt{n}l$$

When an elastomer undergoes reversible deformation under isothermal conditions, its thermodynamic state can be described using the Helmholtz free energy. The Helmholtz free energy is defined as:

(3) A = U − TS

where *A* denotes the Helmholtz free energy, *U* is the internal energy, *T* is the absolute temperature, and *S* is the entropy.

Under isothermal conditions, *dT =* 0, and the differential form can be expressed as:

(4) dA = dU − TdS

If the reversible mechanical work done by the external force on the elastomer is denoted as δW, then:

(5) δW = fdL − PdV

where *f* is the external force in the tensile direction, *L* is the specimen length, *P* is the hydrostatic pressure, and *V* is the volume. For the rubber material used in sealing cylinders, the Poisson’s ratio is close to 0.5; therefore, the material can be approximately regarded as incompressible, and the volume change term *dV* can be neglected. Under this assumption, the tensile force can be obtained from the partial derivative of the Helmholtz free energy with respect to the specimen length:

(6) $$f\;=\;{\left(\frac{\partial A}{\partial L}\right)}_{T}$$

From the perspective of statistical thermodynamics, rubber elasticity mainly originates from changes in the configurational entropy of molecular chains. In the affine network model, three-dimensional deformation characterized by the principal stretch ratios *λ*_1_, *λ*_2_, and *λ*_3_ causes the molecular chains to become more oriented, resulting in a decrease in configurational entropy.

Therefore, the entropy change in the network with a chain density of *N* per unit volume can be written as:

(7) $$∆S\;=\;\frac{1}{2}\mathrm{Nk}\left(\lambda_{1}^{2}\;+\;\lambda_{2\;}^{2}+\;\lambda_{3}^{2}\;-\;3\right)$$

where *k* is the Boltzmann constant. If the change in internal energy is neglected, the strain energy density function can be obtained from *W = −T*Δ*S* as:

(8) $$W\;=\;\frac{1}{2}\mathrm{NkT}\left(\lambda_{1}^{2}\;+{\;\lambda }_{2}^{2}\;+{\;\lambda }_{3}^{2}\;-\;3\right)$$

Here, *k* is the Boltzmann constant, *T* is the absolute temperature, and G = NkT represents the shear modulus. In addition, for incompressible rubber materials, the principal stretches satisfy $\prod_{1}^{3}\lambda_{i}\;=\;1$.

To describe the large-deformation behavior of elastomers from the perspective of continuum mechanics, the deformation gradient tensor needs to be introduced. Let *X* denote the position vector of a material point in the reference configuration, *x = χ(X)* denote its position vector after deformation, and *u = x − X* denote the displacement vector. The deformation gradient can then be expressed as:

(9) $$F_{\mathrm{ij}}\;=\;\frac{\partial x_{i}}{\partial X_{j}}\;=\;\delta_{\mathrm{ij}}\;+\;\frac{\partial u_{i}}{\partial X_{j}},\;J\;\equiv \det F$$

where *δij* is the Kronecker delta, and *J* is the determinant of the deformation gradient tensor.

In the principal coordinate system, the deformation gradient can be written as:

(10) $$F\;=\;\left[\begin{array}{ccc} \lambda_{1} & 0 & 0\\ 0 & \lambda_{2} & 0\\ 0 & 0 & \lambda_{3} \end{array}\right]$$

The left Cauchy–Green deformation tensor is defined as:

(11) $$\begin{array}{c} B\;=\;FF^{T} \end{array}$$

According to the definition of the invariants of the Cauchy–Green deformation tensor, the following relationships can be obtained:

(12) $$I_{1\;}=\;\mathrm{tr}(B)\;={\;B}_{\mathrm{ii}}\;I_{2}\;=\;\frac{1}{2}\left[(\mathrm{trB}{)}^{2}\;-\;\mathrm{tr}\left(B^{2}\right)\right]\;=\;\frac{1}{2}\left(B_{\mathrm{ii}}B_{\mathrm{jj}}\;-\;B_{\mathrm{ij}}B_{\mathrm{ji}}\right)\;I_{3}\;=\;\det(B)\;=\;J^{2}$$

If the deformation is decomposed into volumetric and isochoric parts, the modified invariants can also be defined as:

(13) $${\bar{I}}_{1}\;={\;J}^{\;-2/3}I_{1},\;{\bar{I}}_{2}\;=\;J^{\;-4/3}I_{2}$$

In terms of the principal stretch ratios, the three basic invariants can be expressed as:

(14) $$I_{1}\;=\;\lambda_{1\;}^{2}+\;\lambda_{2}^{2}\;+\;\lambda_{3}^{2}\;\begin{array}{c} I_{2}\;=\;\lambda_{1}^{2}\lambda_{2}^{2}\;+\;\lambda_{2}^{2}\lambda_{3}^{2}\;+\;\lambda_{3}^{2}\lambda_{1}^{2} \end{array}\;I_{3}\;=\;\lambda_{1}^{2}\lambda_{2}^{2}\lambda_{3}^{2}$$

For incompressible materials, *J =* 1, namely *λ*_1_*λ*_2_*λ*_3_ *=* 1. In this case, *I*_3_ *=* 1, and the modified invariants are identical to the corresponding isochoric invariants.

For a hyperelastic material expressed in terms of the principal stretch ratios, if the incompressibility constraint is not considered, the nominal stress in the principal direction can be expressed as:

(15) $$P_{i}\;=\;\frac{\partial W}{\partial \lambda_{i}}$$

The corresponding Cauchy stress in the principal direction is given by:

(16) $$\sigma_{i\;}=\;\frac{\lambda_{i}}{J}\frac{\partial W}{\partial \lambda_{i}}$$

For incompressible hyperelastic materials, a constraint pressure *p* must be introduced. In this case, the Cauchy stress in the principal direction should be written as:

(17) $$\sigma_{i}\;=\;-p\;+\;\lambda_{i}\frac{\partial W}{\partial \lambda_{i}}$$

Therefore, in hyperelastic constitutive modeling of rubber materials, the appropriate stress expression should be selected according to the compressibility of the material, the form of the strain energy density function, and the stress measure adopted.

Elastomer constitutive models are widely used in finite element analysis and provide an important basis for describing the large-deformation mechanical behavior of rubber-like materials. To accurately characterize the nonlinear response of elastomers, various forms of strain energy density functions have been proposed. In practical modeling, the fitting accuracy, number of material parameters, parameter stability, and engineering applicability should be comprehensively considered. Compared with conventional materials such as metals, elastomers usually exhibit more complex mechanical characteristics, including large deformation, viscoelasticity, stress softening, and temperature- and strain-rate-dependent behavior, which makes their constitutive modeling more challenging. Numerical simulation provides an effective approach for investigating the mechanical response of elastomers under geometric and material nonlinearities, and also offers important support for the engineering analysis and optimization design of complex rubber structures.

### 3.2. Hyperelastic Constitutive Modeling of Rubber Materials

Elastomeric materials are characterized by highly nonlinear elastic behavior. Therefore, before performing finite element analysis of practical rubber structures, it is necessary to select an appropriate constitutive model for the specific elastomer under consideration. Several hyperelastic constitutive models, such as the Mooney–Rivlin, Neo-Hookean, Ogden, Yeoh, Arruda–Boyce, and Van der Waals models, can be used to evaluate and describe the mechanical responses of different hyperelastic materials.

#### 3.2.1. Mooney–Rivlin Model [15]

The strain energy density function is expressed as:

(18) $$W=C_{10}({\bar{I}}_{1}\;-\;3)\;+\;C_{01}({\bar{I}}_{2}\;-\;3)$$

where *C*_10_ and *C*_01_ are unknown material parameters, and *J* is the determinant of the deformation gradient *F*, namely *J ≡ detF*.

Under uniaxial mechanical testing conditions, the relationship between the principal stretch ratios and strain can be expressed as:

(19) $$\lambda_{1}\;=\;\lambda_{U},\lambda_{2}\;=\;\lambda_{3}\;={\;\lambda }_{U}^{-1/2},\lambda_{U}\;=\;1\;+\;\varepsilon_{U}$$

The deviatoric strain invariants are expressed as:

(20) $${\bar{I}}_{1}\;=\;\lambda_{U}^{2}\;+\;2\lambda_{U}^{-1},\;{\bar{I}}_{2}\;={\;\lambda }_{U}^{-2}\;+\;2\lambda_{U}$$

The nominal stress–strain relationship is expressed as:

(21) $$T_{U}\;=\;2\left(1\;-{\;\lambda }_{U}^{-3}\right)\left(C_{10}\lambda_{U}\;+\;C_{01}\right)$$

#### 3.2.2. Neo-Hookean Model [16]

The strain energy density function is expressed as:

(22) $$W\;={\;C}_{10}({\bar{I}}_{1}\;-\;3)$$

The nominal stress–strain relationship is expressed as:

(23) $$T_{U}=2C_{10}(\lambda_{U}\;-{\;\lambda }_{U}^{-2})$$

#### 3.2.3. Ogden Model [17]

The strain energy density function is expressed as:

(24) $$\begin{array}{c} W\;=\sum_{i=1}^{N}\frac{2\mu_{i}}{\alpha_{i}^{2}}\left(\lambda_{1}^{\alpha_{i}}\;+\;\lambda_{2}^{\alpha_{i}}\;+\;\lambda_{3}^{\alpha_{i}}\;-\;3\right) \end{array}$$

where $\mu_{i},\;\;\alpha_{i}$ are unknown material parameters, and $\lambda_{i}$ is the principal stretch ratio.

The nominal stress–strain relationship is expressed as:

(25) $$\begin{array}{c} T_{U\;}=\sum_{i=1}^{N}\frac{2\mu_{i}}{\alpha_{i}}\left(\lambda^{\alpha_{i}-1}\;-\;\lambda^{-\frac{1}{2}\alpha_{i}-1}\right) \end{array}$$

#### 3.2.4. Yeoh Model [18]

The strain energy density function is expressed as:

(26) $$\begin{array}{c} W\;=\sum_{i=1}^{3}C_{i0}{\left({\bar{I}}_{1}\;-\;3\right)}^{i} \end{array}$$

where $C_{i0}$ are unknown material parameters.

The nominal stress–strain relationship is expressed as:

(27) $$\begin{array}{cc} T_{U} & =\;2\left(\lambda_{U}\;-\;\lambda_{U}^{-2}\right)C_{10}\;+\;4\left(\lambda_{U}^{3}\;-\;3\lambda_{U}\;+\;3\lambda_{U}^{-2}\;-\;2\lambda_{U}^{-3}\;+\;1\right)C_{20}\\ & +6{\left(\lambda_{U}^{2}\;+\;2\lambda_{U}^{-1}\;-\;3\right)}^{2}\left(\lambda_{U}\;-\;\lambda_{U}^{-2}\right)C_{30} \end{array}$$

#### 3.2.5. Arruda–Boyce Model [19]

The strain energy density function is expressed as:

(28) $$\begin{array}{c} W\;=\;\mu \sum_{i=1}^{5}\frac{C_{i}}{\lambda_{m}^{2i-2}}\left({\bar{I}}_{1}^{i}\;-\;3^{i}\right) \end{array}$$

where $\mu ,\;\lambda_{m}\;$ are unknown material parameters.

The nominal stress–strain relationship is expressed as:

(29) $$\begin{array}{c} T_{U}\;=\;2\mu \left(\lambda_{U}\;-\;\lambda_{U}^{-2}\right)\sum_{i=1}^{5}\frac{iC_{i}}{\lambda_{m}^{2i-2}}{\bar{I}}_{1}^{\;i-1} \end{array}$$

#### 3.2.6. Van Der Waals Model [20]

The strain energy density function is expressed as:

(30) $$\begin{array}{c} W\;=\;\mu \left\{-\left(\lambda_{m}^{2}\;-\;3\right)\left[\ln(1\;-\;\eta )\;+\;\eta \right]\;-\;\frac{2a}{3}{\left(\frac{\tilde{I}\;-\;3}{2}\right)}^{3/2}\right\} \end{array}$$

where

(31) $$\tilde{I}=\left(1-\beta \right){\bar{I}}_{1\;}+\beta {\bar{I}}_{2}$$

(32) $$\eta =\sqrt{\frac{\left(\tilde{I}-3\right)}{\left(\lambda_{m}^{2}-3\right)}}$$

where $\mu ,\;\lambda_{m},\;a\;\mathrm{and}\;\beta$ are unknown material parameters.

The nominal stress–strain relationship is expressed as:

(33) $$\begin{array}{c} T_{U}\;=\;\mu \left(1\;-\;\lambda_{U}^{-3}\right)\left[\frac{1}{1\;-\;\eta }\;-\;a{\left(\frac{\tilde{I}\;-\;3}{2}\right)}^{1/2}\right]\left[\left(1\;-\;\beta \right)\lambda_{U}\;+\;\beta \right] \end{array}$$

### 3.3. Theory of Linear Viscoelasticity

Linear viscoelastic models mainly include the Prony series model, Maxwell model, and Voigt model [21]. Among them, the Prony series model has been most widely used in engineering viscoelastic analysis because of its concise mathematical form, clear physical meaning of parameters, and ease of numerical implementation. It has also been incorporated into various commercial finite element software packages [22].

The Prony series expressed in terms of the dimensionless relaxation modulus is given by:

(34) $$g_{R}\left(t\right)\;=\;\frac{G\left(t\right)}{G_{0}}\;=\;1-\;\sum_{i=1}^{N}{\bar{g}}_{i}^{p}\left[1\;-\;\exp\left(-t/\tau_{i}^{G}\right)\right]$$

where $g_{R}\left(t\right)$ is the relaxation modulus, and N, ${\bar{g}}_{i}^{p}$ and $\tau_{i}^{G}$ are material parameters.

For an isotropic linear elastic material under small-strain conditions, the shear stress can be expressed as:

(35) $$\tau \left(t\right)={\;G}_{0}(\gamma \left(t\right)-\sum_{i=1}^{N}\gamma_{i}\left(t\right))$$

(36) $$\begin{array}{c} \gamma_{i}=\frac{{\bar{g}}_{i}^{p}}{\tau_{i}^{G}}\int_{0}^{t}\exp\left(-\frac{s}{\tau_{i}^{G}}\right)\gamma \left(t-s\right)\mathrm{ds} \end{array}$$

where $G_{0}$ is the instantaneous shear modulus under small-strain conditions, and $\gamma_{i}\left(t\right)$ is the stress relaxation state variable.

The creep strain is defined as the difference between the total strain and the instantaneous elastic strain:

(37) $$\gamma^{\mathrm{cr}}\left(t\right)\;=\sum_{i=1}^{N}\gamma_{i}\left(t\right)$$

### 3.4. Theory of Nonlinear Viscoelasticity

**Figure 3:**
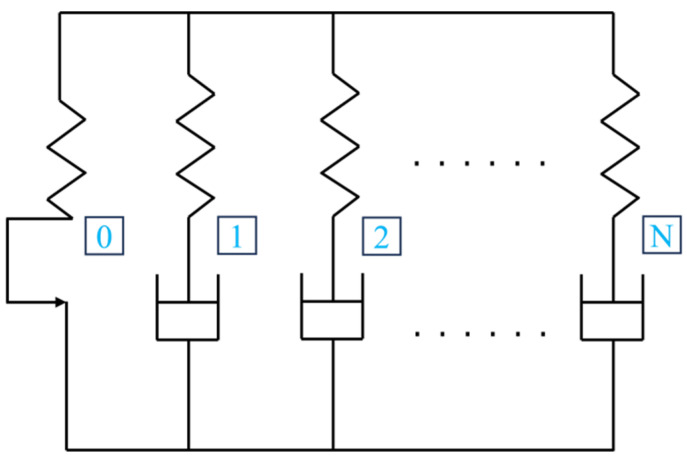
Schematic diagram of the PRF model.

For rubber-like materials, linear viscoelasticity theory alone is insufficient to accurately describe the time-dependent mechanical response under finite deformation. Therefore, a nonlinear constitutive model that can simultaneously account for the coupling between large-deformation effects and viscoelastic evolution is required. The parallel rheological framework (PRF) model decomposes the total material response into an equilibrium elastic response and multiple nonequilibrium viscoelastic responses. Each parallel branch jointly carries the external load and contributes to stress evolution, enabling the nonlinear viscoelastic behavior of hyperelastic materials under complex loading histories to be effectively characterized. The schematic structure of the PRF model is shown in Figure 3.

As shown in Figure 3, the PRF model consists of one equilibrium elastic branch and multiple nonequilibrium viscoelastic branches connected in parallel. The equilibrium branch is used to describe the long-term equilibrium response of the material, whereas the nonequilibrium viscoelastic branches are used to characterize the time-dependent relaxation behavior. The number of viscoelastic branches can be selected according to the stress relaxation characteristics of the material and the required fitting accuracy.

#### 3.4.1. Elastic Response

It is assumed that the elastic response in each network is determined by the hyperelastic strain energy [23]:

(38) $$W\;=\;W_{i}\left(C_{i}^{e}\right)\;\;\;i\;=\;1,\;2,\;\cdots ,\;N$$

where $C_{i}^{e}\;={\;\left(F_{i}^{e}\right)}^{T}$, $F_{i}^{e}$ is the elastic right Cauchy–Green deformation tensor in the i-th network.

It is assumed that all networks share the same form of strain energy function, and the total strain energy *W_T_* is given by the weighted sum of the strain energy contributions from all networks.

(39) $$W_{T}=\sum_{i=1}^{N}S_{i}W\left(C_{i}^{e}\right)$$

where $S_{i}$ represents the stiffness coefficient of the i-th network, namely the volume fraction of that network.

The stiffness coefficients are determined by the material properties and satisfy the following relationship:

(40) $$\sum_{i=1}^{N}S_{i}=\;1$$

The total stress response can be derived from the strain energy function:

(41) $$\tau \;=\;\sum_{i=1}^{N}S_{i}\left\{F_{i}^{e}\cdot 2\frac{\partial W\left(C_{i}^{e}\right)}{\partial \left(C_{i}^{e}\right)}{\left(F_{i}^{e}\right)}^{T}\right\}\;=\;\sum_{i=1}^{N}S_{i}\tau_{i}$$

#### 3.4.2. Viscous Response

For each viscoelastic network, the viscous behavior must be defined separately. By assuming a multiplicative decomposition of the deformation gradient in each network, the deformation gradient can be expressed as:

(42) $$F\;={\;F}_{i}^{e}\;\cdot {\;F}_{i}^{\mathrm{cr}}$$

where $F_{i}^{e}$ represents the elastic part of the deformation gradient, corresponding to the hyperelastic response, while $F_{i}^{\mathrm{cr}}$ represents the creep part of the deformation gradient, associated with the stress-free intermediate configuration. Based on this multiplicative decomposition, the rate form of the creep deformation gradient can be written as:

(43) $$\dot{F_{i}^{\mathrm{cr}}\;}={\;\left(F_{i}^{e}\right)}^{-1}\;\cdot \;D_{i}^{\mathrm{cr}}\;\cdot \;F_{i}^{e}\;\cdot \;F_{i}^{\mathrm{cr}}$$

where $D_{i}^{\mathrm{cr}}$ is the symmetric part of the creep velocity gradient in the *i*-th viscoelastic network.

A flow rule is adopted in which the creep part of the rate-of-deformation tensor is derived from the creep potential $G_{i}^{\mathrm{cr}}$:

(44) $$D_{i}^{\mathrm{cr}}\;=\;{\dot{\lambda }}_{i}\frac{\partial G_{i}^{\mathrm{cr}}}{\partial \tau_{i}}$$

where ${\dot{\lambda }}_{i}$ is the proportionality factor, and $\tau_{i}$ is the Kirchhoff stress tensor of the *i*-th network. In the PRF formulation, the creep potential is commonly defined in terms of the equivalent deviatoric Kirchhoff stress:

(45) $$G_{i}^{\mathrm{cr}}\;=\;q_{i}$$

where

(46) $$q_{i}=\sqrt{\frac{3}{2}s_{i}:s_{i}}$$

and

(47) $$s_{i}=dev\left(\tau_{i}\right)=\tau_{i}-\frac{1}{3}tr\left(\tau_{i}\right)I$$

Here, $s_{i}$ is the deviatoric Kirchhoff stress tensor of the i-th viscoelastic network, and $q_{i}$ is the corresponding equivalent deviatoric Kirchhoff stress.

(48) $$D_{i}^{\mathrm{cr}}={\dot{\bar{\varepsilon }}}_{i}^{cr}\frac{3}{2q_{i}}s_{i}$$

where ${\dot{\bar{\varepsilon }}}_{i}^{cr}$ is the equivalent creep strain rate of the i-th viscoelastic network. In this formulation, the proportionality factor is taken as

(49) $${\dot{\lambda }}_{i}={\dot{\bar{\varepsilon }}}_{i}^{cr}$$

To describe the nonlinear viscous behavior of the material, the strain-hardening creep law is adopted. The equivalent creep strain rate can be expressed as

(50) $${\dot{\bar{\varepsilon }}}_{i}^{cr}={\left(A_{i}\cdot q_{i}^{n_{i}}\cdot {\left[\left(m_{i}+1\right){\bar{\varepsilon }}_{i}^{cr}\right]}^{m_{i}}\right)}^{\frac{1}{m_{i}+1}}$$

where ${\bar{\varepsilon }}_{i}^{cr}$ is the equivalent creep strain, and $A_{i}$, $n_{i}$, and $m_{i}$ are material parameters of the i-th viscoelastic network.

## 4. Finite Element Modeling and Numerical Simulation

**Figure 4:**
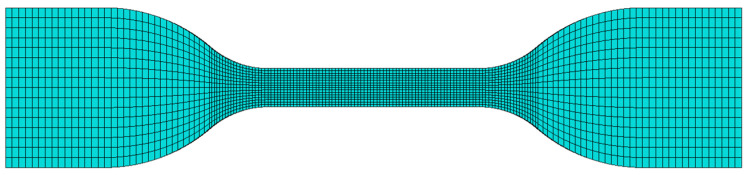
Finite element mesh.

The hyperelastic model parameters and PRF model parameters identified and optimized from the experimental data were used as material inputs, and a finite element model of the rubber tensile specimen was established using ABAQUS 2024. The model was developed to validate the accuracy of the PRF model in describing the nonlinear viscoelastic behavior of rubber materials. The finite element analysis was kept consistent with the stress relaxation test procedure and consisted of two analysis steps. In the first step, an axial displacement of 25 mm was applied to the clamped region at the right end of the rubber specimen, corresponding to a tensile strain of 100%, with a loading time of 15 s. In the second step, the end displacement was kept constant for 1800 s to simulate the stress relaxation process. The finite element mesh is shown in Figure 4. The loading history was defined using an amplitude curve to ensure uniform displacement loading. After the calculation, the stress–time history at the reference point of the finite element model was extracted to obtain the stress–time curve, which was then compared with the experimental results.

## 5. Analysis of Fitting Results

The determination of PRF constitutive model parameters mainly consists of four steps. First, the hyperelastic material parameters are fitted based on the tensile and compressive stress–strain curves. Second, the Prony series parameters are identified using the stress relaxation test data. Third, the Prony series parameters are converted into the initial parameters of the PRF model. Finally, the hyperelastic model parameters and PRF model parameters are iteratively optimized using Isight to obtain material parameters with higher predictive accuracy.

### 5.1. Fitting of Hyperelastic Material Parameters

To systematically evaluate the ability of different hyperelastic constitutive models to describe the large-deformation mechanical behavior of FKM and HNBR materials, two types of rubber with Shore A hardness values of 60, 70, and 80 were selected in this study. Model parameter fitting was carried out based on the experimental data obtained from uniaxial tensile and uniaxial compression tests. To quantitatively assess the fitting accuracy of each model, the coefficient of determination R^2^ was used to evaluate the agreement between the fitted curves and the experimental data. The calculation formula is given as follows:

(51) $$R^{2}=1-\sum_{k=1}^{n}\left(y_{test}-y_{fit}\right)/\sum_{k=1}^{n}\left(y_{test}-\bar{y}\right)$$

where *y_test_* is the experimental value, *y_fit_* is the fitted value, and ȳ is the mean value of the experimental data. A larger *R*^2^ value indicates higher fitting accuracy.

**Figure 5:**
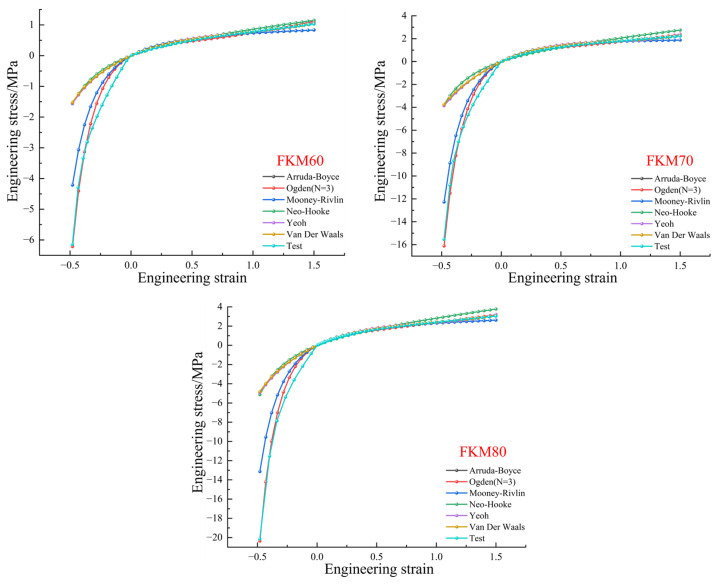
Constitutive fitting curves of FKM rubbers with different hardness levels.

**Figure 6:**
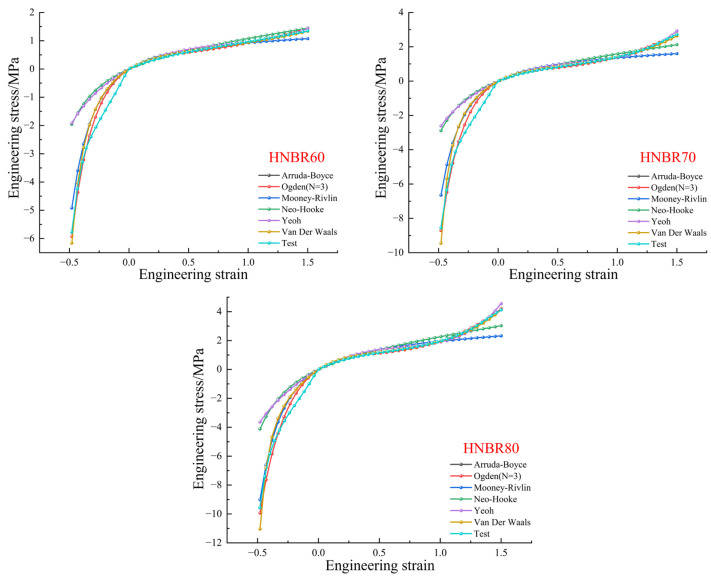
Constitutive fitting curves of HNBR rubbers with different hardness levels.

The coefficient of determination *R*^2^ was calculated to quantitatively evaluate the agreement between each constitutive model and the experimental data. Based on this evaluation, the hyperelastic constitutive model with the highest fitting accuracy was selected to characterize the instantaneous mechanical response of the two rubber materials. The fitting accuracies of different hyperelastic models are listed in Table 1 and Table 2, and the corresponding fitting results are shown in Figure 5 and Figure 6.

The results show that, under the combined experimental conditions of uniaxial tension and compression, the Neo-Hookean model can reasonably reproduce the experimental curves only in the small-strain range, whereas obvious deviations occur in the medium- and large-strain ranges. The Mooney–Rivlin and Yeoh models show good agreement with the experimental results over the full strain range, especially exhibiting stable fitting performance in the medium-strain range. Among all the evaluated models, the Ogden model with N = 3 provides the highest fitting accuracy for large-deformation behavior, with the predicted curves being highly consistent with the experimental data.

Based on the fitting results of six groups of uniaxial mechanical tests, the coefficient of determination *R*^2^ of the Ogden N = 3 model remains at a high level, ranging from 0.9879 to 0.9948. This indicates that the model can accurately and stably describe the nonlinear mechanical response of rubber materials under large deformation. The Arruda–Boyce and Van der Waals models show good performance in the low- and medium-strain ranges, but certain deviations are observed in the high-strain range. Overall, for rubber materials with medium hardness, the Mooney–Rivlin and Yeoh models can achieve acceptable accuracy while maintaining computational efficiency, making them suitable for engineering calculations. In contrast, the Ogden N = 3 model exhibits the best fitting performance in the large-deformation range and is therefore more suitable for high-accuracy finite element simulations. The fitted constitutive model parameters are listed in Table 3.

### 5.2. Fitting of Prony Series Material Parameters

To obtain the relevant parameters of the Prony series, the stress relaxation experimental data of fluororubber (FKM) and hydrogenated nitrile rubber (HNBR) with hardness levels of 60, 70, and 80 were normalized. Specifically, the stress value $\sigma_{t}$ at each time point was divided by the initial stress $\sigma_{0}$ to obtain the normalized stress relaxation modulus curve.

The Prony series was used to identify the parameters of the normalized stress relaxation curve. Let the normalized relaxation modulus be *E(t)*; its expression in the time domain can be written as:

(52) $$E\left(t\right)=E_{\infty }+\sum_{i=1}^{n}E_{i}\;\exp\left(-\frac{t}{\tau_{i}}\right)$$

where $E_{\infty }$ is the long-term relaxation modulus, $E_{i}$ is the modulus coefficient of the i-th Prony term, $\tau_{i}$ is the corresponding relaxation time, and *n* is the number of Prony series terms.

The instantaneous modulus can then be obtained as:

(53) $$E_{0}\;=\;E_{\infty }+\sum_{i=1}^{n}E_{i}$$

**Figure 7:**
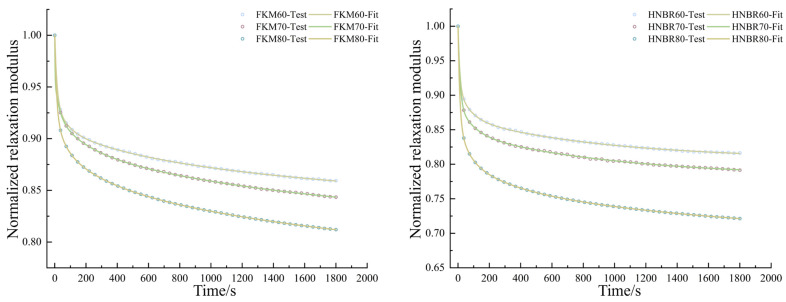
Fitting of stress relaxation data using the Prony series method.

The dimensionless coefficients required for finite element implementation were then further calculated. The normalized stress relaxation modulus curves and the corresponding fitting curves are shown in Figure 7.

(54) $$\begin{array}{c} g_{i}=\frac{E_{i}}{E_{0}} \end{array}$$

The normalized relaxation curves of both rubber materials exhibit typical viscoelastic characteristics, namely a rapid decrease in the early stage followed by a slow variation in the later stage. This indicates that the molecular chain segments inside the material rearrange rapidly at the initial stage of loading and then gradually enter a stable relaxation stage. For the FKM materials in Figure 7, the curves corresponding to different hardness levels are clearly separated, with the normalized relaxation response following the order FKM80 < FKM70 < FKM60. This suggests that the normalized stress retention capability decreases and the degree of stress relaxation increases with increasing hardness. Similarly, for the HNBR materials in Figure 7, the curves also follow the order HNBR80 < HNBR70 < HNBR60, indicating that the relaxation degree of HNBR also increases with hardness. A further comparison between the FKM and HNBR curves in Figure 7 shows that, within the same time range, the HNBR curves are generally lower than the corresponding FKM curves at the same hardness level, indicating that HNBR exhibits greater stress decay and weaker long-term stress retention capability than FKM. The fitted Prony series parameters of FKM and HNBR rubbers with different hardness levels are shown in Table 4.

**Figure 8:**
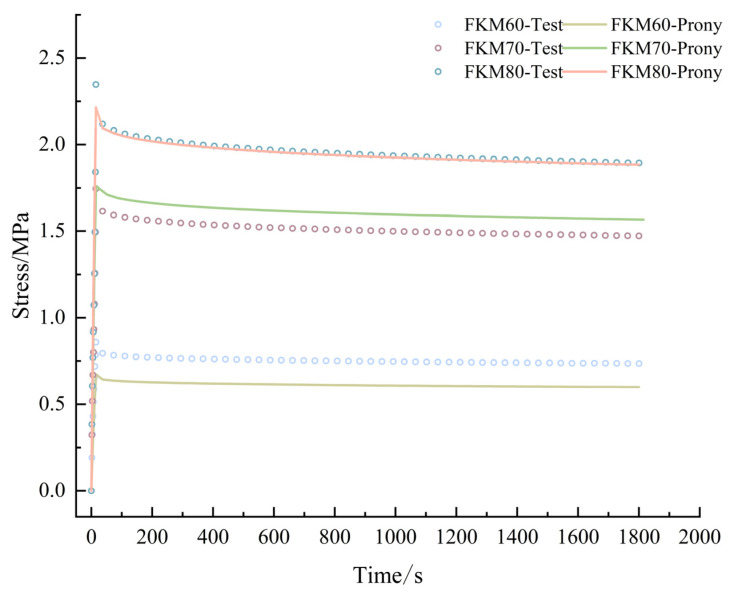
Comparison between Prony series fitting results and experimental stress relaxation data for FKM rubber.

As shown in Figure 8, the three FKM rubbers with different hardness levels exhibit typical stress relaxation behavior during the holding stage. The stress decreases rapidly at the initial stage, followed by a gradual reduction in the relaxation rate, and eventually tends to stabilize. In addition, the stress level increases with increasing hardness, following the order FKM80 > FKM70 > FKM60. By comparing the experimental results with the finite element results, it can be observed that the finite element model is capable of capturing the general trend of stress relaxation. However, the fitting accuracy varies among the different hardness levels. Specifically, the simulated results for FKM80 are in relatively good agreement with the experimental data, whereas the calculated stress of FKM60 is generally lower than the experimental value. For FKM70, the simulated stress is slightly higher than the experimental result in the middle and later stages of relaxation. These results indicate that the current Prony series parameters still lead to certain deviations in describing the relaxation behavior of the material, particularly in terms of uniformly characterizing the relaxation amplitude and stress level under different hardness conditions.

**Figure 9:**
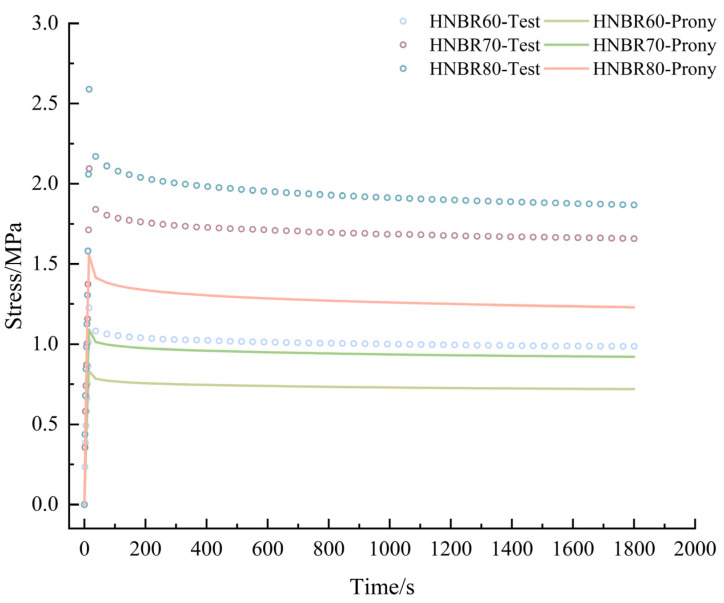
Comparison between Prony series fitting results and experimental stress relaxation data for HNBR.

As shown in Figure 9, HNBR60, HNBR70, and HNBR80 all exhibit typical stress relaxation behavior during the holding stage. The stress decreases rapidly at the initial stage, after which the relaxation rate gradually decreases and the stress response tends to stabilize in the later stage. Meanwhile, both the experimental and finite element results show that the overall stress level increases with increasing hardness, following the order HNBR80 > HNBR70 > HNBR60. This indicates that increasing the hardness of HNBR enhances its load-bearing capacity and residual stress level.

A comparison between the experimental and finite element curves shows that the finite element model can reasonably capture the overall stress relaxation trend and the relative stress level among the three hardness grades. However, noticeable deviations remain in the predicted stress magnitude, with the finite element results generally lower than the experimental results. Among the three materials, the simulated response of HNBR60 is relatively close to the experimental data, whereas larger deviations are observed for HNBR70 and HNBR80. In particular, the experimental curves remain significantly higher than the simulated curves throughout the relaxation stage. This suggests that although the finite element model established using the fitted viscoelastic parameters can describe the time-dependent behavior of HNBR, it is still insufficient to accurately characterize the actual relaxation amplitude and stress retention capability under different hardness conditions.

### 5.3. Conversion of Prony Series Material Parameters into Linear PRF Constitutive Parameters

In this study, the PRF model with a power-law strain hardening formulation was adopted to describe the time-dependent viscoelastic behavior of rubber materials. In the linear PRF model, the parameters were set as (m = 0) and (n = 1), under which the model can be correlated with the Prony series formulation. Therefore, the Prony series parameters were mapped into the PRF power-law strain hardening model to obtain the initial material parameters of the linear PRF model. The corresponding parameters are listed in Table 5 and Table 6. The conversion relationships between the Prony series parameters and the linear PRF model parameters are given as follows:

(55) $${\mathrm{SR}}_{i}\;=\;g_{i}$$

(56) $$\begin{array}{c} A_{i}=1/\left(6\times g_{i}\times \tau_{i}\times \mu_{0}\right) \end{array}$$

**Figure 10:**
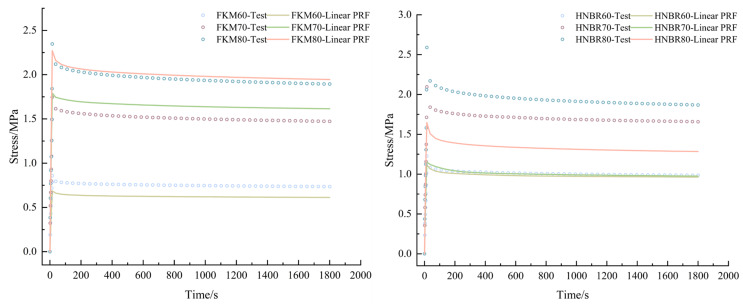
Comparison between linear PRF fitting results and experimental stress relaxation data for the two rubber materials.

The calculated results of the linear PRF model were compared with the experimental data, as shown in Figure 10. The results indicate that although the linear PRF model can preliminarily capture the stress relaxation trend of the material, certain deviations from the experimental curves remain, making it insufficient to accurately describe the nonlinear viscoelastic response of rubber materials. Therefore, in this study, the parameters of the linear PRF model were further used as initial values, and the PRF model parameters were iteratively optimized using the Isight 2024 data matching method to improve the predictive accuracy of the model with respect to the experimental data.

### 5.4. Isight-Based Optimization of PRF Material Parameters

**Figure 11:**
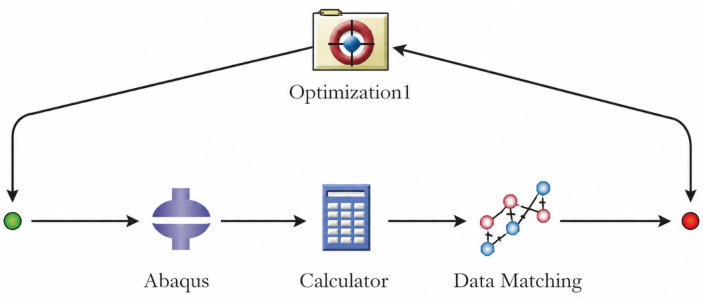
Flowchart of Isight-based optimization of PRF material parameters.

The material parameters of the nonlinear PRF model were optimized using the Data Matching component in Isight 2024, and the optimization workflow is shown in Figure 11. First, the previously identified hyperelastic model parameters and linear PRF model parameters were used as the initial inputs to establish the Abaqus finite element model for stress relaxation analysis. Subsequently, the stress–time history data at the reference point of the finite element model were extracted, and the error between the simulated results and the experimental stress relaxation data was calculated using the Calculator component. Finally, the PRF model parameters were iteratively updated using the Data Matching component until the stress relaxation curve obtained from the finite element simulation achieved the best agreement with the experimental curve. During the optimization process, the Hooke–Jeeves algorithm was adopted for parameter searching. This algorithm is a gradient-free direct search method and is particularly suitable for parameter optimization problems in finite element simulations where the objective function gradient is difficult to obtain explicitly. By continuously adjusting the PRF model parameters and minimizing the discrepancy between the simulation results and the experimental data, the nonlinear PRF viscoelastic constitutive parameters capable of accurately describing the stress relaxation behavior of rubber materials were finally obtained. The optimized hyperelastic parameters and PRF model parameters of the rubber materials are listed in Table 7.

**Figure 12:**
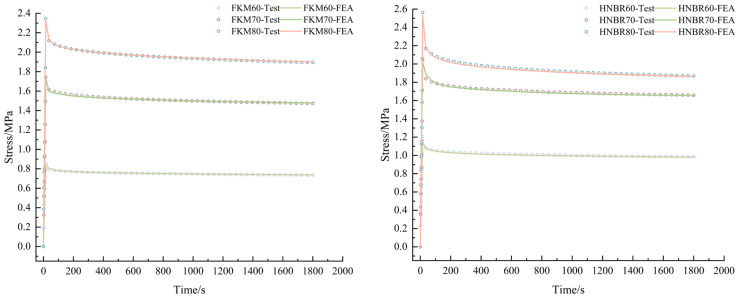
Comparison between nonlinear PRF fitting results and experimental stress relaxation data for the two rubber materials.

The stress–time history data at the reference point in the finite element model were extracted and compared with the experimentally measured stress relaxation data. The resulting stress relaxation curves of HNBR and FKM over a testing duration of 1800 s are shown in Figure 12. By comparing the experimental relaxation data with the finite element simulation results obtained from the nonlinear PRF model, it can be observed that the two sets of curves show good overall agreement for both FKM and HNBR. This indicates that the viscoelastic constitutive model established based on the nonlinear PRF has good predictive accuracy.

To further quantitatively evaluate the agreement between the model predictions and the experimental data, the mean absolute percentage error (MAPE) was used to assess the prediction accuracy of the nonlinear PRF model. For the FKM materials, the MAPE values of FKM60, FKM70, and FKM80 were 2.67%, 1.57%, and 2.56%, respectively. For the HNBR materials, the MAPE values of HNBR60, HNBR70, and HNBR80 were 2.16%, 2.72%, and 2.58%, respectively. Overall, the MAPE values of both rubber materials were lower than 3%, and the error levels among different hardness grades were relatively close. These results demonstrate that the nonlinear PRF model can accurately describe the stress relaxation behavior of FKM and HNBR rubbers within 1800 s and exhibits good applicability to rubber materials with different hardness levels. Based on the curve comparison and quantitative error analysis, the nonlinear PRF model can effectively characterize the nonlinear viscoelastic relaxation response of rubber materials with high computational accuracy and reliability.

## 6. Conclusions

In this study, the nonlinear hyper-viscoelastic mechanical behavior of FKM and HNBR materials under large-deformation and stress relaxation conditions was investigated. Hyperelastic model fitting, Prony series parameter identification, PRF parameter conversion, Isight-based parameter optimization, and finite element validation were systematically carried out. A constitutive parameter identification procedure for describing the nonlinear viscoelastic response of rubber materials was established. The main conclusions are as follows:

(1) Different hyperelastic constitutive models exhibit significant differences in their ability to describe the large-deformation mechanical behavior of rubber materials. The Neo-Hookean model has a simple formulation with few parameters, but it is mainly applicable to the small-strain range. The Mooney–Rivlin and Yeoh models show good stability and engineering applicability in the moderate-strain range. In contrast, the Ogden model with (N = 3) provides the highest fitting accuracy under large-deformation conditions. Based on the fitting results of six groups of FKM and HNBR rubbers with different hardness levels, the coefficient of determination (R2) of the Ogden (N = 3) model ranges from 0.9879 to 0.9948. This indicates that the Ogden (N = 3) model can accurately and stably describe the nonlinear large-deformation response of rubber materials and is more suitable for high-accuracy finite element simulations.

(2) Both FKM and HNBR exhibit typical viscoelastic characteristics during the 1800 s stress relaxation process. The stress decreases rapidly at the initial stage and then gradually approaches a stable state with a reduced relaxation rate. As the hardness increases, the overall stress level of the material increases, whereas the normalized stress retention capability decreases, indicating that higher-hardness rubbers show a greater relative degree of stress relaxation while maintaining higher load-bearing capacity. A comparison between the two rubber types shows that, at the same hardness level, HNBR exhibits a larger stress decay than FKM, suggesting that FKM has better long-term stress retention capability.

(3) The Prony series can describe the general trend of rubber stress relaxation curves; however, it is essentially a linear viscoelastic model and has limited capability in characterizing the nonlinear viscoelastic response of rubber materials under finite deformation. After converting the Prony series parameters into the initial parameters of the linear PRF model, the linear PRF model can preliminarily reproduce the relaxation trend, but noticeable deviations from the experimental curves remain. This indicates that relying solely on the Prony series or linear PRF parameters is insufficient to accurately characterize the actual relaxation amplitude and stress retention behavior of rubber materials with different hardness levels.

(4) By using the linear PRF parameters as initial values and further optimizing the nonlinear PRF model parameters through the Isight data matching method, the agreement between the finite element stress relaxation curves and the experimental curves is significantly improved. The error analysis shows that the mean absolute percentage errors of FKM60, FKM70, and FKM80 are 2.67%, 1.57%, and 2.56%, respectively, while those of HNBR60, HNBR70, and HNBR80 are 2.16%, 2.72%, and 2.58%, respectively. The MAPE values of both rubber types are below 3%, demonstrating that the optimized nonlinear PRF model can accurately describe the stress relaxation behavior of FKM and HNBR rubbers with different hardness levels and exhibits good stability and applicability.

(5) Overall, the combination of the Ogden (N = 3) model for describing the instantaneous large-deformation response and the optimized nonlinear PRF model for characterizing the time-dependent viscoelastic response provides a comprehensive description of the nonlinear hyper-viscoelastic behavior of rubber materials. The proposed method offers a reliable material modeling basis for finite element analysis, constitutive parameter identification, and structural optimization of rubber sealing components, vibration isolation elements, damping components, and sealing rubber cylinders used in pipeline plugging robots.

## Figures and Tables

**Table 1:** Fitting accuracy of hyperelastic constitutive models for FKM rubber.

Shore Hardness/Degree	Hyperelastic Constitutive Model	*R* ^2^
60	Ogden, N = 3	0.9886
	Mooney–Rivlin	0.9123
	Yeoh	0.5794
	Van der Waals	0.5723
	Arruda–Boyce	0.5641
	Neo-Hookean	0.5641
70	Ogden, N = 3	0.9924
	Mooney–Rivlin	0.9597
	Yeoh	0.5768
	Van der Waals	0.5656
	Arruda–Boyce	0.5358
	Neo-Hookean	0.5358
80	Ogden, N = 3	0.9948
	Mooney–Rivlin	0.9008
	Yeoh	0.5668
	Van der Waals	0.5581
	Arruda–Boyce	0.5601
	Neo-Hookean	0.5601

**Table 2:** Fitting accuracy of hyperelastic constitutive models for HNBR.

Shore Hardness/Degree	Hyperelastic Constitutive Model	*R* ^2^
60	Ogden, N = 3	0.9879
	Mooney–Rivlin	0.9592
	Yeoh	0.6949
	Van der Waals	0.9735
	Arruda–Boyce	0.6799
	Neo-Hookean	0.6799
70	Ogden, N = 3	0.9907
	Mooney–Rivlin	0.9296
	Yeoh	0.6878
	Van der Waals	0.9669
	Arruda–Boyce	0.6950
	Neo-Hookean	0.6950
80	Ogden, N = 3	0.9922
	Mooney–Rivlin	0.9534
	Yeoh	0.7902
	Van der Waals	0.9774
	Arruda–Boyce	0.7931
	Neo-Hookean	0.7931

**Table 3:** Material parameters of the hyperelastic constitutive model.

Rubber Materials	Shore Hardness/Degree	Group	Material Parameters	
			μ	α
FKM	60	1	−208.372	0.365
		2	97.511	0.584
		3	111.607	0.141
	70	1	−432.3	0.278
		2	199.075	0.5
		3	235.26	0.0507
	80	1	−309.512	−0.0906
		2	141.739	0.1894
		3	170.139	−0.37772
HNBR	60	1	−202.792	0.923
		2	94.529	1.154
		3	109.183	0.686
	70	1	−336.006	1.278
		2	156.176	1.53
		3	181.159	1.019
	80	1	−391.735	1.814
		2	181.025	2.073
		3	212.623	1.547

**Table 4:** Fitted Prony series parameters of FKM and HNBR rubbers with different hardness levels.

Rubber Materials	Shore Hardness/Degree	Group	$g_{i}$	$K_{i}$	$\tau_{i}$
FKM	60	1	0.06162	0	13.348
		2	0.02569	0	61.925
		3	0.01778	0	337.042
		4	0.05989	0	1963.684
	70	1	0.03006	0	187.434
		2	0.03307	0	30.755
		3	0.07307	0	1636.771
		4	0.04469	0	1.899
	80	1	0.03073	0	52.337
		2	0.08544	0	1937.466
		3	0.03266	0	254.19
		4	0.07302	0	11.103
HNBR	60	1	0.05977	0	907.137
		2	0.04253	0	76.48
		3	0.02499	0	179,985
		4	0.08998	0	10.822
	70	1	1.174 × 10^−13^	0	179,546.4
		2	0.0415	0	93.702
		3	0.06462	0	974.577
		4	0.112	0	12.722
	80	1	0.1368	0	10.836
		2	0.03976	0	258.043
		3	0.07938	0	1632.304
		4	0.04909	0	58.961

**Table 5:** Conversion between Prony series parameters and linear PRF parameters for FKM.

Shore Hardness/Degree	Prony Series		Power-Law Strain Hardening Model	
60	$\mu_{0}=\mu_{1}+\mu_{2}+\mu_{3}$	0.746	m	0
			n	1
	$g_{1}$	0.06162	${\mathrm{SR}}_{1}$	0.06162
	$\tau_{1}$	13.348	$A_{1}$	0.27163
	$g_{2}$	0.02569	${\mathrm{SR}}_{2}$	0.02569
	$\tau_{2}$	61.925	$A_{2}$	0.14044
	$g_{3}$	0.01778	${\mathrm{SR}}_{3}$	0.01778
	$\tau_{3}$	337.042	$A_{3}$	0.03728
	$g_{4}$	0.05989	${\mathrm{SR}}_{4}$	0.05989
	$\tau_{4}$	1963.684	$A_{4}$	0.0019
70	$\mu_{0}=\mu_{1}+\mu_{2}+\mu_{3}$	2.035	m	0
			n	1
	$g_{1}$	0.03006	${\mathrm{SR}}_{1}$	0.03006
	$\tau_{1}$	187.434	$A_{1}$	0.01454
	$g_{2}$	0.03307	${\mathrm{SR}}_{2}$	0.03307
	$\tau_{2}$	30.755	$A_{2}$	0.08053
	$g_{3}$	0.07307	${\mathrm{SR}}_{3}$	0.07307
	$\tau_{3}$	1636.771	$A_{3}$	0.00068
	$g_{4}$	0.04469	${\mathrm{SR}}_{4}$	0.04469
	$\tau_{4}$	1.899	$A_{4}$	0.96505
80	$\mu_{0}=\mu_{1}+\mu_{2}+\mu_{3}$	2.366	m	0
			n	1
	$g_{1}$	0.03073	${\mathrm{SR}}_{1}$	0.03073
	$\tau_{1}$	52.337	$A_{1}$	0.0438
	$g_{2}$	0.08544	${\mathrm{SR}}_{2}$	0.08544
	$\tau_{2}$	1937.466	$A_{2}$	0.000426
	$g_{3}$	0.03266	${\mathrm{SR}}_{3}$	0.03266
	$\tau_{3}$	254.19	$A_{3}$	0.00849
	$g_{4}$	0.07302	${\mathrm{SR}}_{4}$	0.07302
	$\tau_{4}$	11.103	$A_{4}$	0.08689

**Table 6:** Conversion between Prony series parameters and linear PRF parameters for HNBR.

Shore Hardness/Degree	Prony Series		Power-Law Strain Hardening Model	
60	$\mu_{0}=\mu_{1}+\mu_{2}+{\;\mu }_{3}$	0.92	m	0
			n	1
	$g_{1}$	0.05977	${\mathrm{SR}}_{1}$	0.05977
	$\tau_{1}$	907.137	$A_{1}$	0.0033412
	$g_{2}$	0.04253	${\mathrm{SR}}_{2}$	0.04253
	$\tau_{2}$	76.48	$A_{2}$	0.0557
	$g_{3}$	0.02499	${\mathrm{SR}}_{3}$	0.02499
	$\tau_{3}$	179,985	$A_{3}$	0.0000403
	$g_{4}$	0.08998	${\mathrm{SR}}_{4}$	0.08998
	$\tau_{4}$	10.822	$A_{4}$	0.186
70	$\mu_{0}={\;\mu }_{1}+\mu_{2}+\mu_{3}$	1.329	m	0
			n	1
	$g_{1}$	1.174 × 10^−13^	${\mathrm{SR}}_{1}$	1.174 × 10^−13^
	$\tau_{1}$	1.79546 × 10^5^	$A_{1}$	1.826486 × 10^6^
	$g_{2}$	0.0415	${\mathrm{SR}}_{2}$	0.0415
	$\tau_{2}$	93.702	$A_{2}$	0.0099
	$g_{3}$	0.06462	${\mathrm{SR}}_{3}$	0.06462
	$\tau_{3}$	974.577	$A_{3}$	0.000611
	$g_{4}$	0.112	${\mathrm{SR}}_{4}$	0.112
	$\tau_{4}$	12.722	$A_{4}$	0.02702
80	$\mu_{0}=\mu_{1}+\mu_{2}+\mu_{3}$	1.913	m	0
			n	1
	$g_{1}$	0.1368	${\mathrm{SR}}_{1}$	0.1368
	$\tau_{1}$	10.836	$A_{1}$	0.05877
	$g_{2}$	0.03976	${\mathrm{SR}}_{2}$	0.03976
	$\tau_{2}$	258.043	$A_{2}$	0.00849
	$g_{3}$	0.07938	${\mathrm{SR}}_{3}$	0.07938
	$\tau_{3}$	1632.304	$A_{3}$	0.00067
	$g_{4}$	0.04909	${\mathrm{SR}}_{4}$	0.04909
	$\tau_{4}$	58.961	$A_{4}$	0.0301

**Table 7:** Optimized hyperelastic parameters and PRF model parameters of FKM and HNBR rubber materials.

Rubber Materials	Shore Hardness/Degree	Group	$\mu_{i}$	$\alpha_{i}$	${\mathrm{SR}}_{i}$	$A_{i}$	$n_{i}$	$m_{i}$
FKM	60	1	−207.96	0.36573	0.062945	0.2647	1.025	−0.025
		2	97.218	0.58575	0.026242	0.13939	1.0155	0.0205
		3	111.61	0.14192	0.018162	0.038082	0.979	0.0205
		4	/	/	0.061178	0.0019408	0.979	0.0205
	70	1	−432.73	0.27772	0.030108	0.014546	0.9995	0.0005
		2	199.37	0.49925	0.033123	0.08057	0.9996	0.0009
		3	235.26	0.050619	0.073187	6.8109 × 10^−4^	0.9985	0.0011
		4	/	/	0.044757	0.96602	0.9985	0.0009
	80	1	−309.51	−0.09046	0.030791	0.043362	1.01	−0.0095
		2	141.74	0.18845	0.087149	4.352 × 10^−4^	0.9805	0.02
		3	170.14	−0.37961	0.033166	8.4051 × 10^−3^	1.0025	0.001
		4	/	/	0.073385	0.088628	0.984	−0.009
HNBR	60	1	−202.59	0.92208	0.05971	0.0033379	1.001	−0.001
		2	94.529	1.1552	0.042487	0.055644	1.001	−0.001
		3	109.18	0.68669	0.024965	4.03 × 10^−5^	1.001	−0.001
		4	/	/	0.08989	0.18581	1.001	−0.001
	70	1	−336.01	1.2461	1.174 × 10^−13^	1,826,500	1.0	0.0
		2	156.96	1.4841	0.0442	0.010593	0.93	−0.04
		3	181.16	0.99352	0.06882	6.5377 × 10^−4^	0.93	−0.02
		4	/	/	0.11984	0.028911	0.93	−0.01
	80	1	−391.34	1.8122	0.13615	0.059049	0.99525	−0.0345
		2	181.02	2.0771	0.039571	0.0084497	1.0048	−0.005
		3	212.62	1.547	0.079	6.6682 × 10^−4^	1.0048	−0.005
		4	/	/	0.048857	0.030017	1.0022	−0.005

## Data Availability

The data supporting the findings of this study solely comprise original data. Due to confidentiality constraints, these data cannot be made publicly available. Further inquiries can be directed to the corresponding author. The datasets used or analysed during the current study are available from the corresponding author on reasonable request.
